# Supplementary material for: Collagen Structured Hydration
Source: Biomolecules. 2023 Dec 4;13(12):1744. doi: 10.3390/biom13121744 (PMC10742079; doi:10.3390/biom13121744)
Supplement: Supplementary file 1 [file biomolecules-13-01744-s001.zip › biomolecules-2619285-supplementary.pdf]

Supplementary Documents

Collagen Structured Hydration

Satyaranjan Biswal, Noam Agmon*

Institute of Chemistry, Hebrew University of Jerusalem.

Table of contents:

Table S1: Average root mean square deviation (RMSD) for the collagen-like peptide $[(PPG)_{10}]_3$ in aqueous solution at two different temperatures, 300 K and 250 K [AMBER14SB+TIP4P/2005, 62306 WMs, 100 ns simulation (10000 frames)].

Table S2: Comparison of the average end to end distance for five collagen mimetic peptides, $[(PPG)_n]_3$, n = 4, 5, 7, 9, and 10, in aqueous solution at two different temperatures, 300 K and 250 K.

Table S3: Position of the RDF maxima and minima, r in Å, for water surrounding GLY-CO or PRO(Y)-CO, in the collagen-like peptide $[(PPG)_{10}]_3$ [AMBER14SB+TIP4P/2005, 62306 WMs, 100 ns simulation (10000 frames)].

Table S4: (A-C). (Capped protein) Solvent accessible surface area (SASA, in Å^2) for the bb-carbonyl oxygen system for the three chains of the collagen-like peptide $[(PPG)_{10}]_3$ at 300 K [AMBER14SB+TIP4P/2005, 62306 WMs, 100 ns simulation (10000 frames)].

Table S5: (A-C). (Capped protein) Solvent accessible surface area (SASA, in Å^2) for the bb-carbonyl oxygen system in the thee chains of the collagen-like peptide $[(PPG)_{10}]_3$ at 250 K [AMBER14SB+TIP4P/2005, 62306 WMs, 100 ns simulation (10000 frames)].

Figure S1: The time dependence of the root mean square displacement (RMSD) for the collagen-like peptide $[(PPG)_{10}]_3$ at three different temperatures, 300 K (red) 250 K (black) and 100 K (blue). [AMBER14SB+TIP4P/2005, 62306 WMs, 100 ns simulation (10000 frames)].

Figure S2: The time dependence of the radius of gyration (Rg) for the collagen-like peptide $[(PPG)_{10}]_3$ at three different temperatures, 300 K (red) 250 K (black) and 100 K (blue) [AMBER14SB+TIP4P/2005, 62306 WMs, 100 ns simulation (10000 frames)].

Figure S3: The number of water molecules hydrogen-bonded to the backbone carbonyl (bb-CO) sites in the crystal structure of $[(PPG)_9]_3$ (PDB ID -2CUO), as a function of residue number.

Figure S4: Oxygen–oxygen radial distribution function for liquid water at T = 300 K for the TIP4P/2005 (red) and TIP3P (black) water models, in comparison with the experimental results of Skinner et al., *J. Chem Phys.* 138, 074506 (2013), Fig. 9(a), at T = 298 K.

Figure S5: Comparison of the RDF profiles of water around the carbonyl oxygen of the Gly and Pro (Y) residues of $[(PPG)_{10}]_3$ for the TIP4P/2005 (red) and TIP3P (black) water models

(both with the AMBER14SB FF and 10,000 saved frames), at 300 and 250 K [TIP4P/2005, 62306 WMs; TIP3P, 61801 WMs].

Figure S6: Comparison of the RDF profiles of water around the carbonyl oxygen of the Gly and Pro (Y) residues of $[(PPG)_{10}]_3$ for two different FFs, CHARMM36m (black) and AMBER14SB (red), both with the TIP4P/2005 water model [T = 300 K, 62306 WMs, 10000 frames].

Figure S7: Number of water neighbors in the first solvation shell of the Res-CO sites on all three chains of the $[(PPG)_{10}]_3$ peptide, obtained from integrating g(r) at two different temperatures, 250 K (black) and 300 K (red) [AMBER14SB+TIP4P/2005, 62306 WMs, 100 ns simulation (10000 frames)]. This figure extends Figure 8.

Figure S8: Number of water neighbors in the first solvation shell of the Res-CO [where Res = (A) Gly and (B) Pro (Y)] sites on the all three chains of the $[(PPG)_{10}]_3$ peptide, obtained from integrating g(r) at two different temperatures, 250 K (black) and 300 K (red) [AMBER14SB+TIP4P/2005, 62306 WMs, 100 ns simulation (10000 frames)].

Figure S9: Comparison of the number of water neighbors in the first solvation shell of the Res-CO sites on all three chains of the $[(PPG)_{10}]_3$ peptide, for the TIP4P/2005 (black line) and TIP3P (red line) water models, obtained from integrating g(r) from Figure S5. [TIP4P/2005 (62306 WMs), TIP3P (61801 WMs), 10000 frames].

Figure S10: Solvent accessible surface area (SASA, in Å^2) for the bb-carbonyl oxygen atoms of all three chains of the $[(PPG)_{10}]_3$ peptide at 250 K (black) and 300 K (red) [AMBER14SB+TIP4P/2005, 62306 WMs, 100 ns simulation, 10000 frames]. This extends Figure 9.

Figure S11: Solvent accessible surface area (SASA, in Å^2) for the bb-carbonyl oxygen atoms of all three chains residues of the $[(PPG)_{10}]_3$ peptide at 250 K (black) and 300 K (red) (A) Gly and (B) Pro (Y) [AMBER14SB+TIP4P/2005, 62306 WMs, 100 ns simulation (10000 frames)].

Figure S12: Solvent accessible surface area (SASA, in Å^2) for the bb-carbonyl oxygen atoms of all three chains residues of the $[(PPG)_{10}]_3$ for the TIP3P water model (black) in comparison with (red) [TIP4P/2005,62306 WMs, TIP3P (61801 WMs), 10000 frames].

Figure S13: Correlation plot of the number of water neighbors with the oxygen carbonyl SASA values in our simulations of collagen triple-helix at 250 K [AMBER14SB+TIP4P/2005, 62306 WMs, 100 ns trajectory (10000 frames)]. To be compared with Figure 10 at 300 K.

Tcl Scripts.

Tcl script: S1 Average number of neighbors for each atom in a given selection

Tcl script: S2 Calculate SASA for each residue or atom of a selection

Tcl script: S3 Residence time and its distribution

Tcl script: S4 RMSD: Measures average RMSD & std for given selections in a trajectory

Tcl script: S5 RMSF (Root Mean Square Fluctuation) in a trajectory

Tcl script: S6 Radius of gyration (without the mass scaling)

Tcl script: S7 Measures distance, average distance & std, for any given 2 atoms

Table S1: Average root mean square deviation (RMSD) for the collagen-like peptide $[(PPG)_{10}]_3$ in aqueous solution at two different temperatures, 300 K and 250 K [AMBER14SB+TIP4P/2005, 62306 WMs, 100 ns simulation (10000 frames)].

Temperature	RMSD [Å]
300 K	1.95
250 K	1.77
100 K	0.27

Table S2: Comparison of the average end to end distance for five collagen mimetic peptides, $[(PPG)_n]_3$, n = 4, 5, 7, 9, and 10, in aqueous solution at two different temperatures, 300 K and 250 K.

SYSTEM	(Average end to end distance) $[(PPG)_9]_3$	(Standard deviation) $[(PPG)_9]_3$	(Average end to end distance) $[(PPG)_{10}]_3$	(Standard deviation) $[(PPG)_{10}]_3$
Chain-A (300 K)	72.71	1.14	81.50 (A)	1.06
Chain-B (300 K)	72.59	1.17	81.49 (A)	1.01
Chain-C (300 K)	72.17	1.23	81.24 (A)	1.08
Chain-A (250 K)	72.64	0.83	81.45 (A)	0.93
Chain-B (250 K)	72.50	1.02	81.47 (A)	0.89
Chain-C (250 K)	72.09	1.00	81.22 (A)	0.97

SYSTEM	(Average end to end distance) $[(PPG)_7]_3$	(Standard deviation) $[(PPG)_7]_3$	(Average end to end distance) $[(PPG)_5]_3$	(Standard deviation) $[(PPG)_5]_3$
Chain-A (300 K)	54.07	1.38	38.98	0.94
Chain-B (300 K)	55.02	1.15	39.06	0.78
Chain-C (300 K)	54.12	1.02	38.49	0.95
Chain-A (250 K)	54.01	0.85	38.40	0.91
Chain-B (250 K)	55.01	0.80	39.02	0.66
Chain-C (250 K)	54.09	0.94	38.51	0.77

SYSTEM	(Average end to end distance) $[(PPG)_4]_3$	(Standard deviation) $[(PPG)_4]_3$	(Average end to end distance) $[(PPG)_3]_3$	(Standard deviation) $[(PPG)_3]_3$
Chain-A (300 K)	32.96	0.96	23.07	0.61
Chain-B (300 K)	32.93	0.92	23.11	0.55
Chain-C (300 K)	32.46	1.00	22.76	0.54
Chain-A (250 K)	32.59	0.93	23.06	0.57
Chain-B (250 K)	32.61	0.91	23.02	0.52
Chain-C (250 K)	32.43	0.96	22.75	0.57

Table S3: Position of the RDF maxima and minima, r in Å, for water surrounding GLY-CO or PRO(Y)-CO, in the collagen-like peptide $[(PPG)_{10}]_3$ [AMBER14SB+TIP4P/2005, 62306 WMs, 100 ns simulation (10000 frames)].

Systems	r 1st maxima [Å]	r 2nd maxima [Å]	r 1st minima [Å]	r 2nd minima [Å]
GLY-CO-- O_W (250 K)	2.65	4.35	3.15	5.65
GLY-CO-- O_W (300 K)	2.65	4.45	3.25	5.55
PRO(Y)-CO-- O_W (250 K)	2.65	4.35	3.25	5.55
PRO(Y)-CO-- O_W (300 K)	2.65	4.35	3.25	5.55

Table S4: (A-C). (Capped protein) Solvent accessible surface area (SASA, in Å^2) for the bb-carbonyl oxygen system for the three chains of the collagen-like peptide $[(PPG)_{10}]_3$ at 300 K [AMBER14SB+TIP4P/2005, 62306 WMs, 100 ns simulation (10000 frames)].

Table-4A (Chain -A)

**Res_i**	Gly3	Pro4	Pro5	Gly6	Pro7	Pro8	Gly9	Pro10	Pro11	Gly12	Pro13	Pro14	Gly15
**SASA**	6.99	0.003	13.18	6.61	0.006	13.20	6.31	0.004	13.30	6.23	0.007	13.77	6.32
**Res_i**	Pro16	Pro17	Gly18	Pro19	Pro20	Gly21	Pro22	Pro23	Gly24	Pro25	Pro26	Gly27	Pro28
**SASA**	0.007	14.19	6.78	0.007	14.15	6.98	0.004	13.54	6.82	0.006	13.25	16.73	6.14

Table-4B (Chain -B)

**Res_i**	Gly3	Pro4	Pro5	Gly6	Pro7	Pro8	Gly9	Pro10	Pro11	Gly12	Pro13	Pro14	Gly15
**SASA**	6.33	0.03	13.48	6.32	0.004	13.75	6.33	0.004	14.02	6.83	0.005	14.42	6.84
**Res_i**	Pro16	Pro17	Gly18	Pro19	Pro20	Gly21	Pro22	Pro23	Gly24	Pro25	Pro26	Gly27	Pro28
**SASA**	0.004	13.42	7.11	0.003	13.25	6.36	0.005	13.41	6.41	0.012	13.23	6.09	0.42

Table-4C (Chain -C)

**Res$_i$**	Gly3	Pro4	Pro5	Gly6	Pro7	Pro8	Gly19	Pro10	Pro11	Gly12	Pro13	Pro14	Gly16
**SASA**	6.18	0.010	13.98	6.69	0.005	14.21	6.84	0.003	13.69	7.04	0.003	13.28	6.36
**Res$_i$**	Pro16	Pro17	Gly18	Pro19	Pro20	Gly21	Pro22	Pro23	Gly24	Pro25	Pro26	Gly27	Pro28
**SASA**	0.008	13.43	6.32	0.003	13.50	6.34	0.005	13.77	6.42	0.005	14.21	6.40	0.052

Table S5: (A-C). (Capped protein) Solvent accessible surface area (SASA, in Å^2) for the bb-carbonyl oxygen system in the thee chains of the collagen-like peptide [(PPG)$_{10}$]$_3$ at 250 K [AMBER14SB+TIP4P/2005, 62306 WMs, 100 ns simulation (10000 frames)].

Table-5A (Chain -A)

**Res$_i$**	Gly3	Pro4	Pro5	Gly6	Pro7	Pro8	Gly9	Pro10	Pro11	Gly12	Pro13	Pro14	Gly15
**SASA**	6.70	0.266	12.40	6.35	0.001	12.63	6.06	0.003	12.75	6.05	0.001	13.43	6.07
**Res$_i$**	Pro16	Pro17	Gly18	Pro19	Pro20	Gly21	Pro22	Pro23	Gly24	Pro25	Pro26	Gly27	Pro28
**SASA**	0.004	14.33	6.48	0.001	13.41	6.56	0.001	12.47	6.94	0.003	15.68	6.26	0.58

Table-5B (Chain -B)

**Res$_i$**	Gly3	Pro4	Pro5	Gly6	Pro7	Pro8	Gly9	Pro10	Pro11	Gly12	Pro13	Pro14	Gly15
**SASA**	6.08	0.001	11.63	6.08	0.003	13.55	6.35	0.001	15.69	6.76	0.007	13.06	6.52
**Res$_i$**	Pro16	Pro17	Gly18	Pro19	Pro20	Gly21	Pro22	Pro23	Gly24	Pro25	Pro26	Gly27	Pro28
**SASA**	0.002	14.37	6.22	0.026	13.66	6.84	0.005	12.42	6.74	0.008	12.40	7.02	0.256

Table-5C (Chain -C)

Res_i	Gly3	Pro4	Pro5	Gly6	Pro7	Pro8	Gly9	Pro10	Pro11	Gly12	Pro13	Pro14	Gly15
**SASA**	6.62	0.032	15.96	6.54	0.001	14.18	6.29	0.004	13.33	5.97	0.003	13.88	6.09
**Res_i**	Pro16	Pro17	Gly18	Pro19	Pro20	Gly21	Pro22	Pro23	Gly24	Pro25	Pro26	Gly27	Pro28
**SASA**	0.001	12.77	5.72	0.002	12.48	6.36	0.007	12.70	6.09	0.004	12.78	6.75	0.038

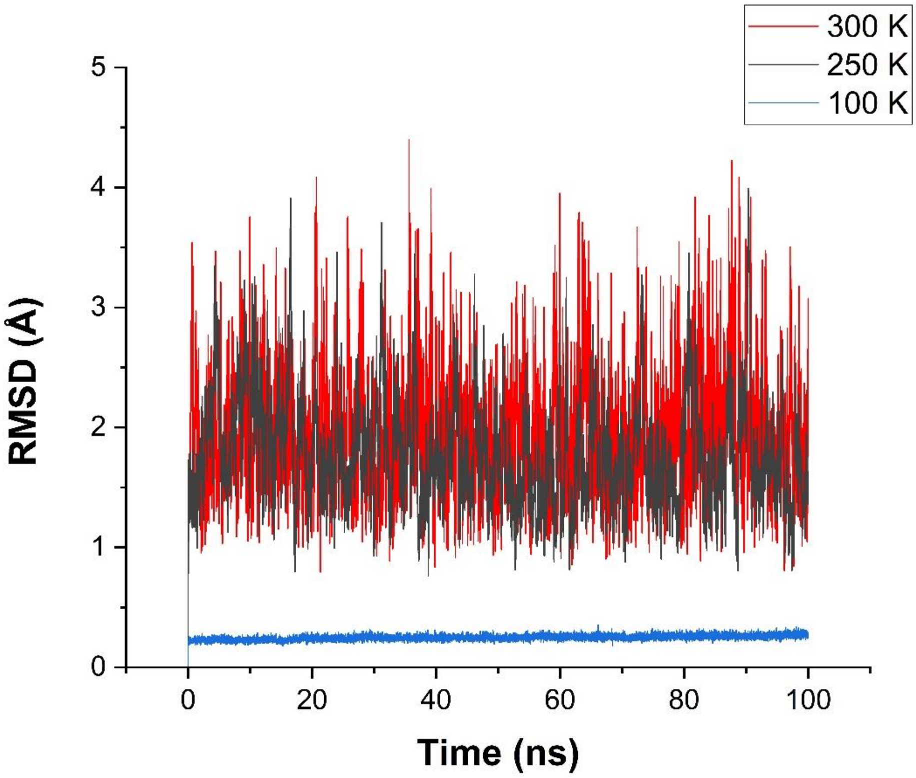

Figure S1: The time dependence of the root mean square displacement (RMSD) for the collagen-like peptide $[(PPG)_{10}]_3$ at three different temperatures, 300 K (red) 250 K (black) and 100 K (blue). [AMBER14SB+TIP4P/2005, 62306 WMs, 100 ns simulation (10000 frames)].

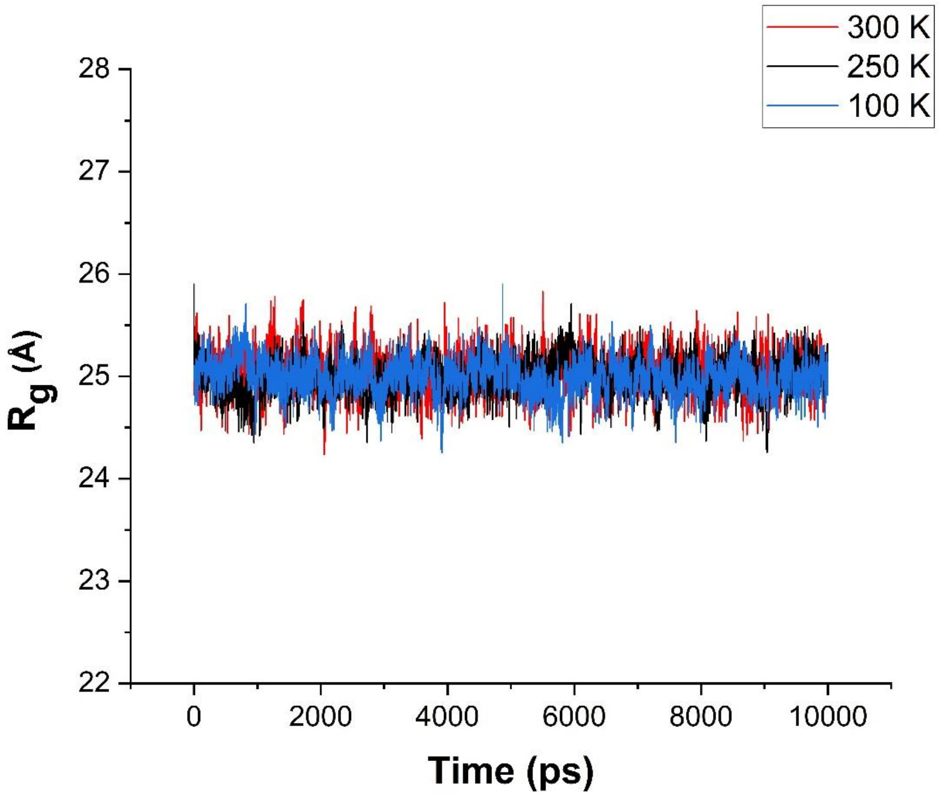

Figure S2: The time dependence of the radius of gyration (Rg) for the collagen-like peptide $[(PPG)_{10}]_3$ at three different temperatures, 300 K (red) 250 K (black) and 100 K (blue) [AMBER14SB+TIP4P/2005, 62306 WMs, 100 ns simulation (10000 frames)].

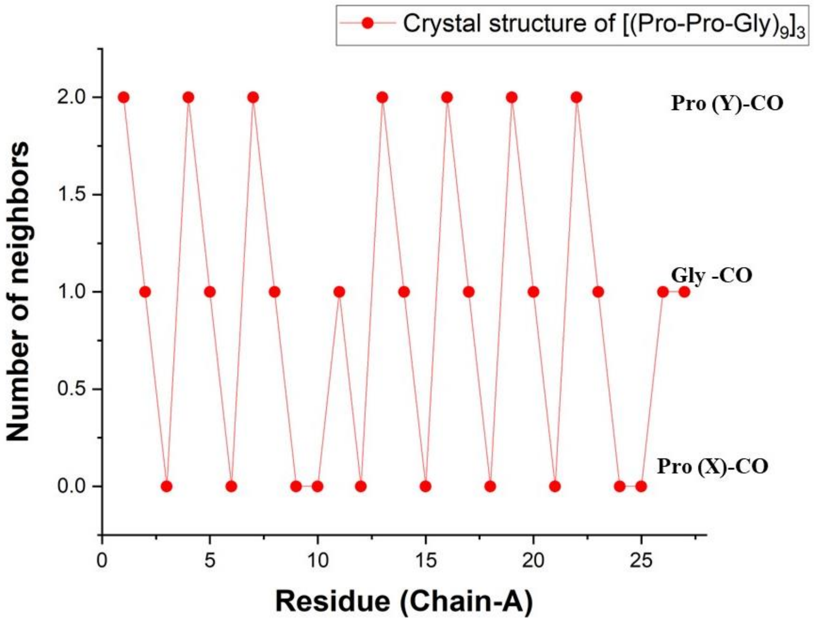

Figure S3: The number of water molecules hydrogen-bonded to the backbone carbonyl (bb-CO) sites in the crystal structure of chain α of $[(PPG)_9]_3$ (PDB ID = 2CUO, 1.33 Å resolution), as a function of residue number. Compare with Figure 5.

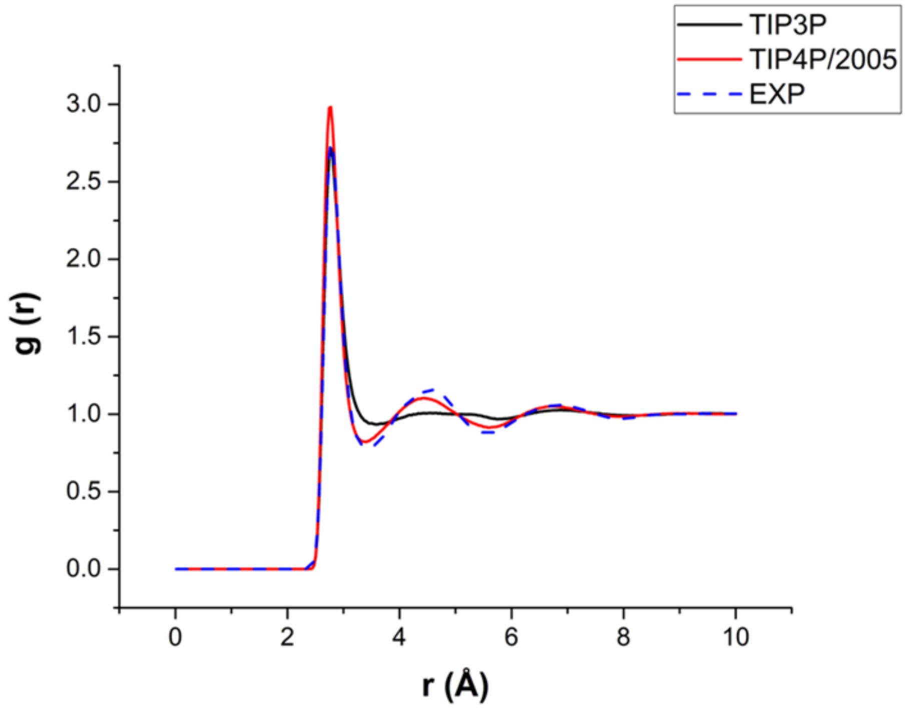

Figure S4: Oxygen–oxygen radial distribution function for liquid water at T = 300 K for the TIP4P/2005 (red) and TIP3P (black) water models, in comparison with the experimental results of LB Skinner et al., *J. Chem Phys.* 138, 074506 (2013), Fig. 9(a), at T = 298 K.

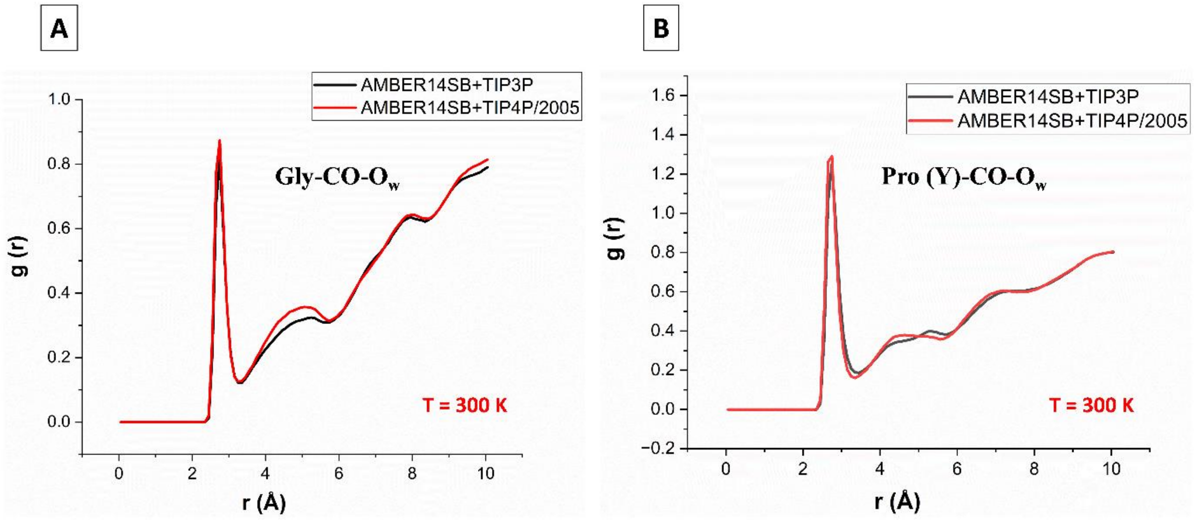

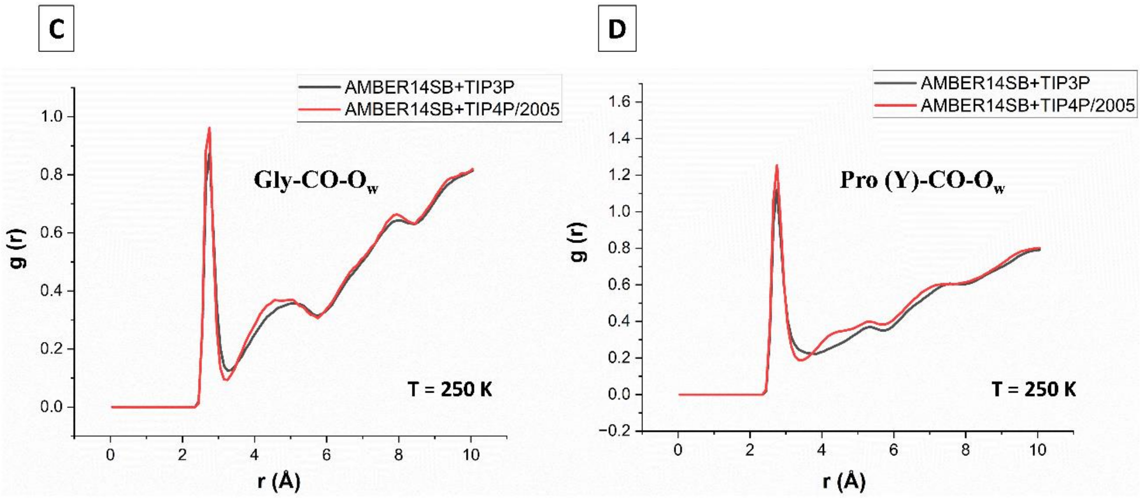

Figure S5: Comparison of the RDF profiles of water around the carbonyl oxygen of the Gly (A, C) and Pro (Y) residues (B, D) of $[(PPG)_{10}]_3$ for the TIP4P/2005 (red) and TIP3P (black) water models (both with the AMBER14SB FF and 10,000 saved frames), at 300 K (A, B) and 250 K (C,D) [TIP4P/2005, 62306 WMs; TIP3P, 61801 WMs].

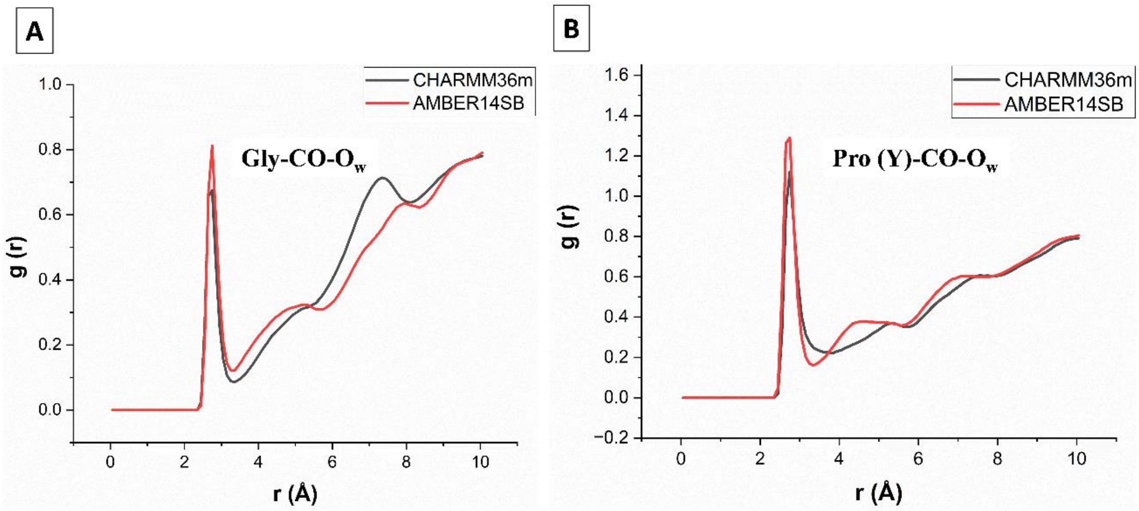

Figure S6: Comparison of the RDF profiles of water around the carbonyl oxygen of the Gly (A) and Pro (Y) residues (B) of $[(PPG)_{10}]_3$ for two different FFs, CHARMM36m (black) and AMBER14SB (red), both with the TIP4P/2005 water model [T = 300 K, 62306 WMs, 10000 frames].

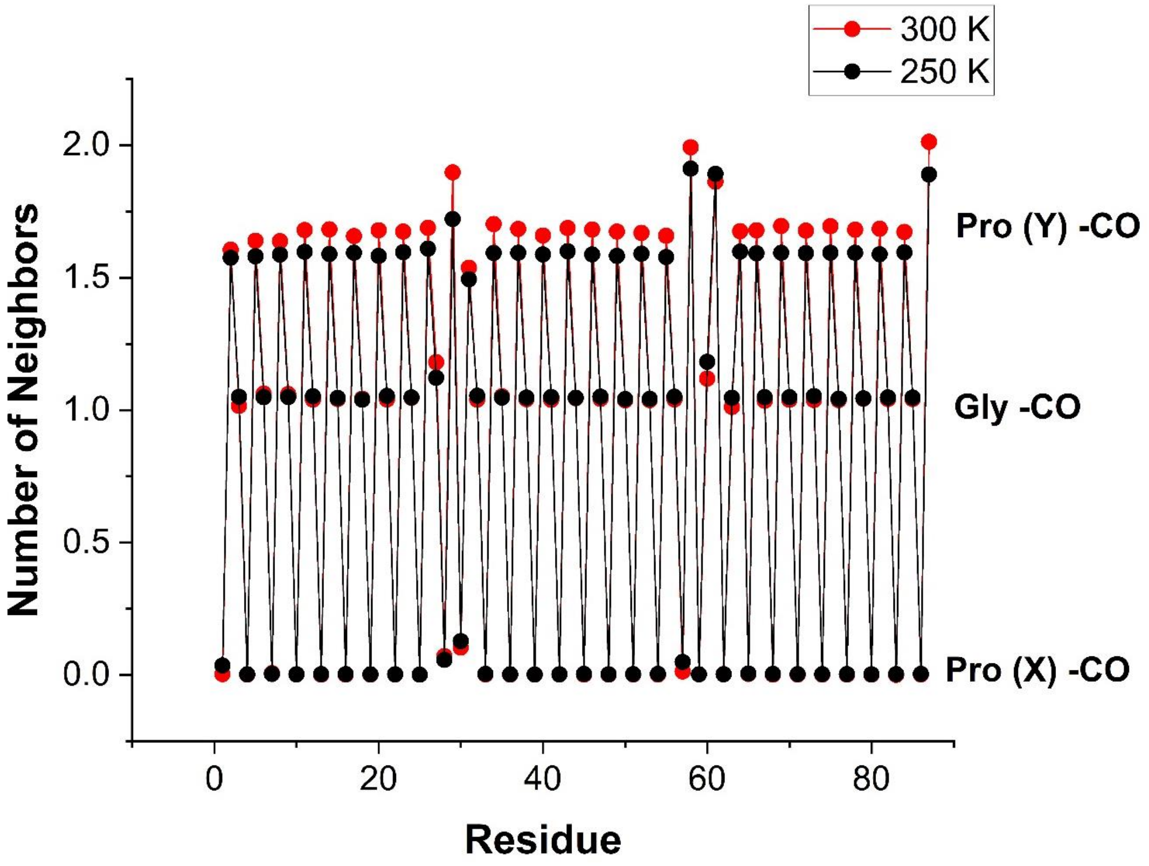

Figure S7: Number of water neighbors in the first solvation shell of the Res-CO sites on all three chains of the $[(PPG)_{10}]_3$ peptide, obtained from integrating g(r) at two different temperatures, 250 K (black) and 300 K (red) [AMBER14SB+TIP4P/2005, 62306 WMs, 100 ns simulation (10000 frames)]. This figure extends Figure 8.

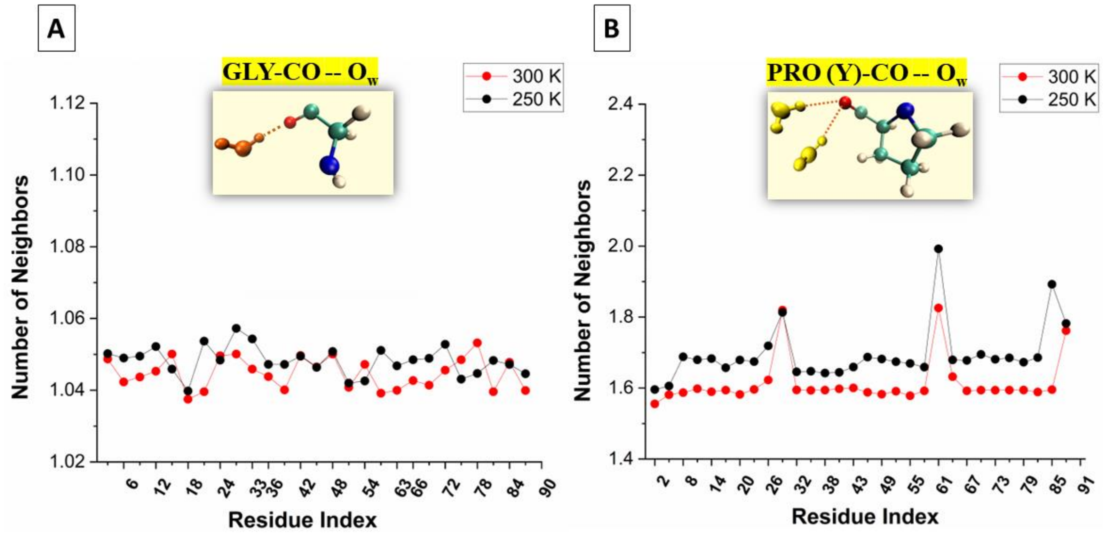

Figure S8: Number of water neighbors in the first solvation shell of the Res-CO [where Res = (A) Gly and (B) Pro (Y)] sites on the all three chains of the $[(PPG)_{10}]_3$ peptide, obtained from integrating g(r) at two different temperatures, 250 K (black) and 300 K (red) [AMBER14SB+TIP4P/2005, 62306 WMs, 100 ns simulation (10000 frames)].

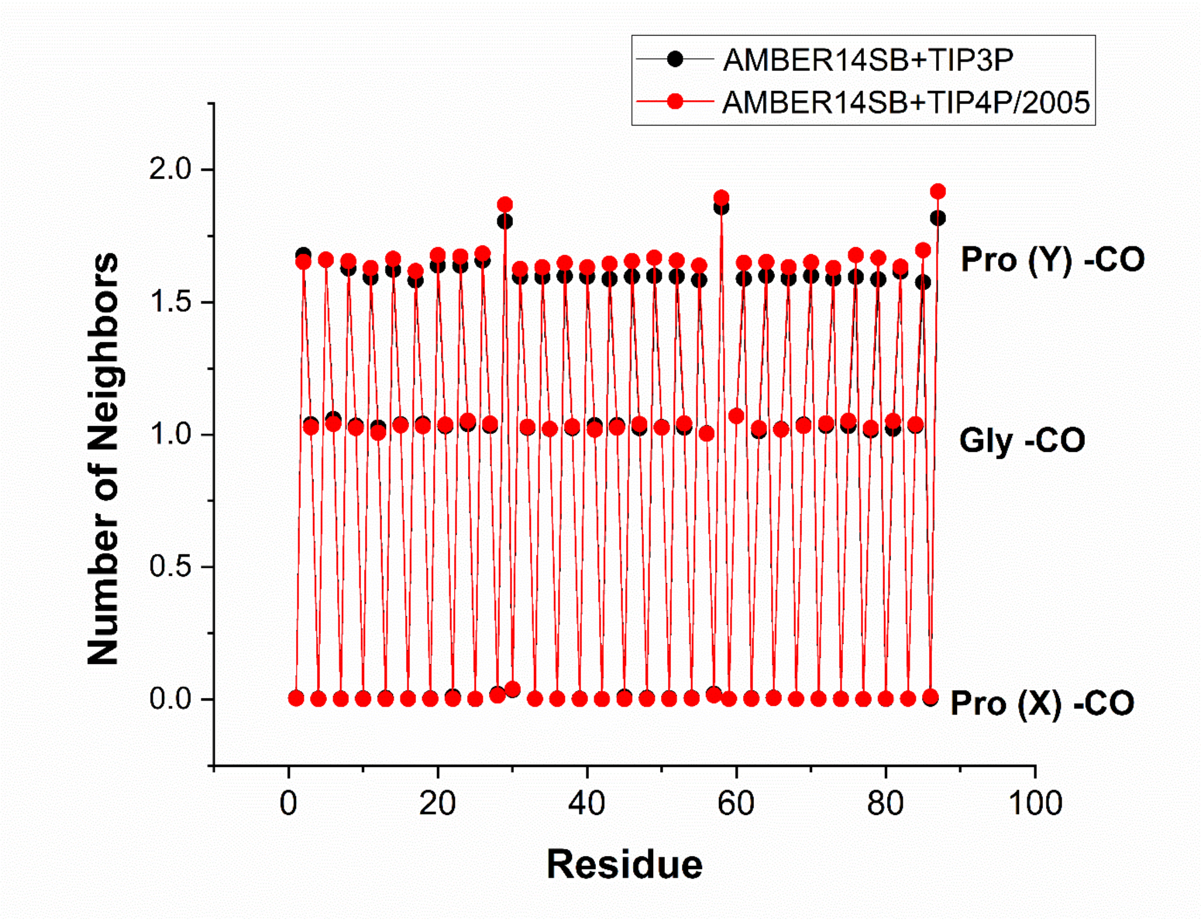

Figure S9: Comparison of the number of water neighbors in the first solvation shell of the Res-CO sites on all three chains of the $[(PPG)_{10}]_3$ peptide, for the TIP4P/2005 (red line) and TIP3P (black line) water models, obtained from integrating g(r) from Figure S5. [T=300 K, TIP4P/2005 (62306 WMs), TIP3P (61801 WMs), 10000 frames].

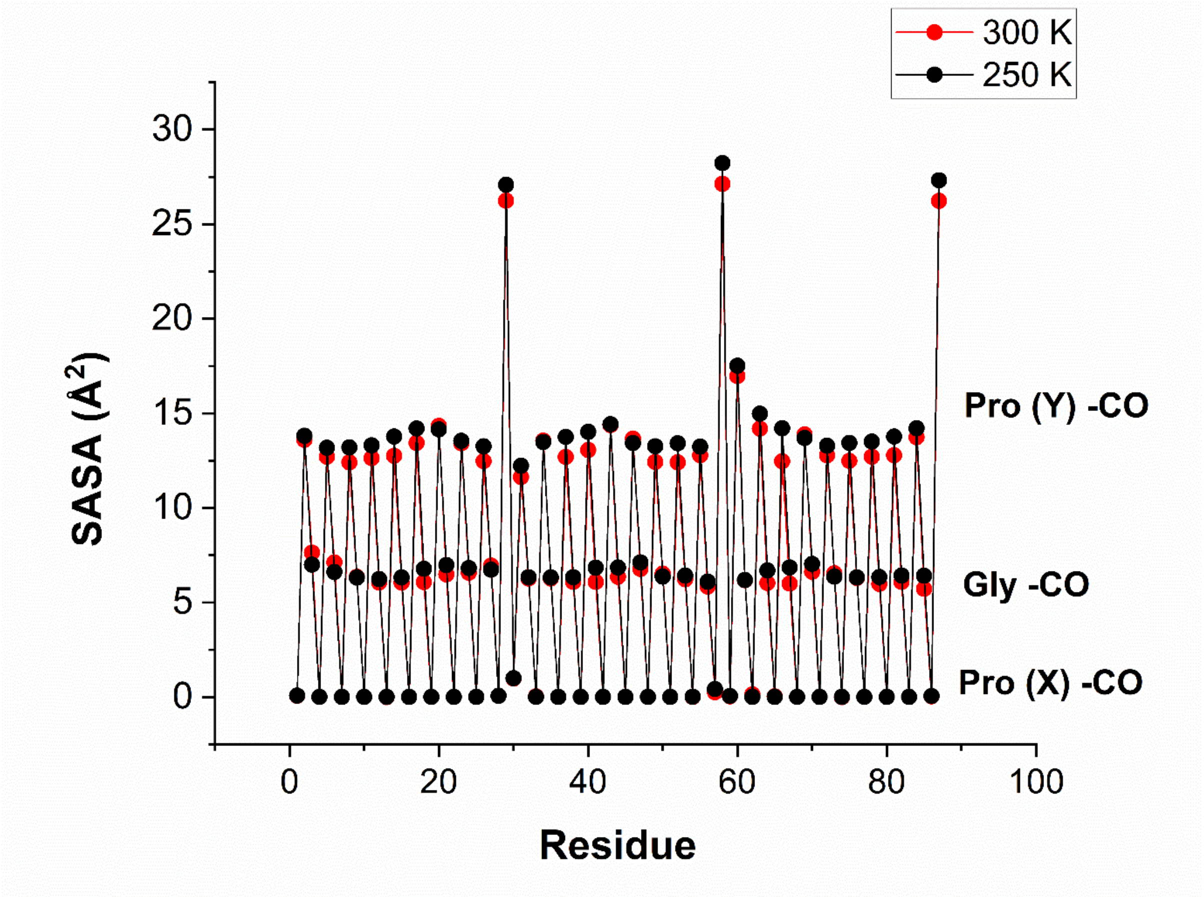

Figure S10: Solvent accessible surface area (SASA, in Å^2) for the bb-carbonyl oxygen atoms of all three chains of the [(PPG)$_{10}$]$_3$ peptide at 250 K (black) and 300 K (red) [AMBER14SB+TIP4P/2005, 62306 WMs, 100 ns simulation, 10000 frames]. This extends Figure 9.

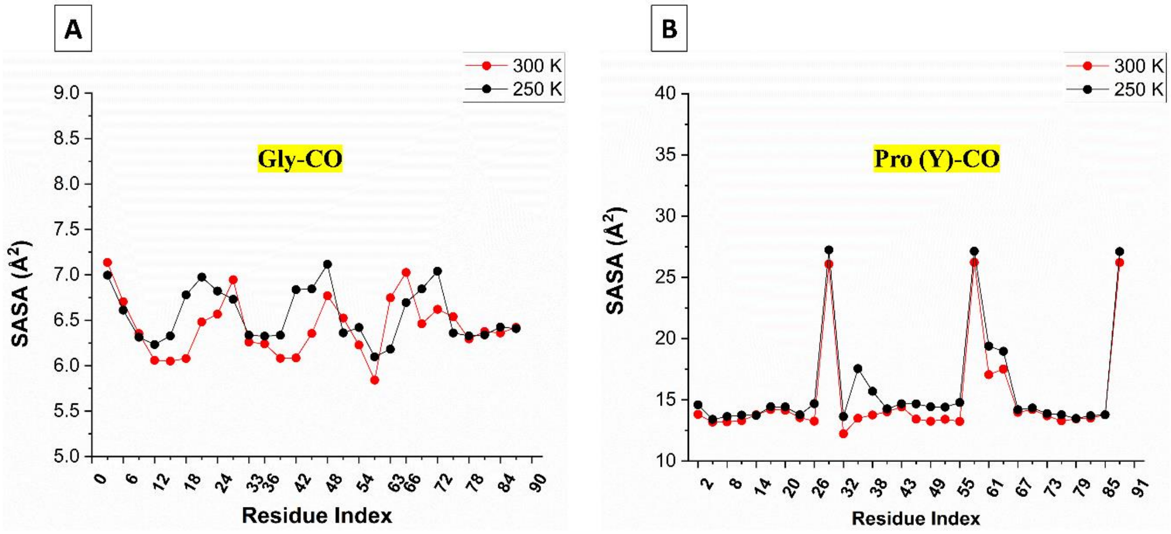

Figure S11: Solvent accessible surface area (SASA, in Å^2) for the bb-carbonyl oxygen atoms of all three chains residues of the [(PPG)$_{10}$]$_3$ peptide at 250 K (black) and 300 K (red) (A) Gly and (B) Pro (Y) [AMBER14SB+TIP4P/2005, 62306 WMs, 100 ns simulation (10000 frames)].

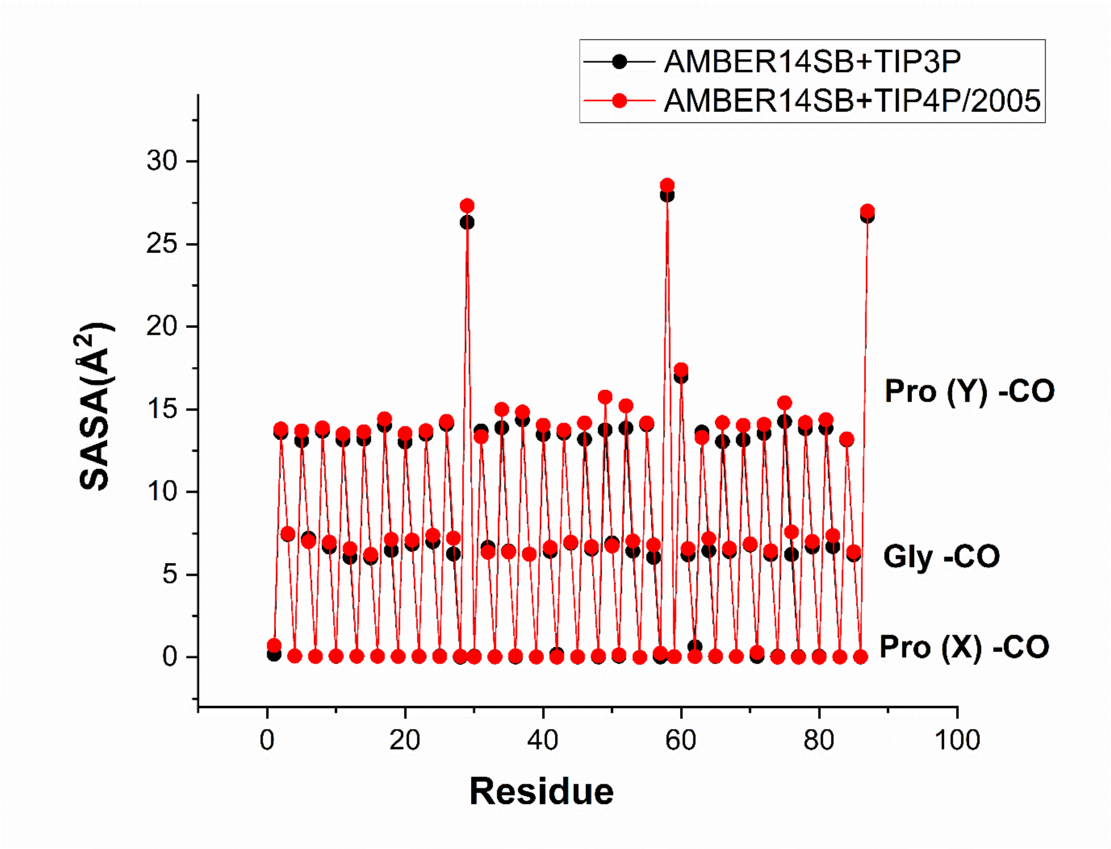

Figure S12: Solvent accessible surface area (SASA, in Å^2) for the bb-carbonyl oxygen atoms of all three chains residues of the [(PPG)$_{10}$]$_3$ for the TIP3P water model (black) in comparison with (red) [T=300 K, TIP4P/2005 (62306 WMs), TIP3P (61801 WMs), 10000 frames].

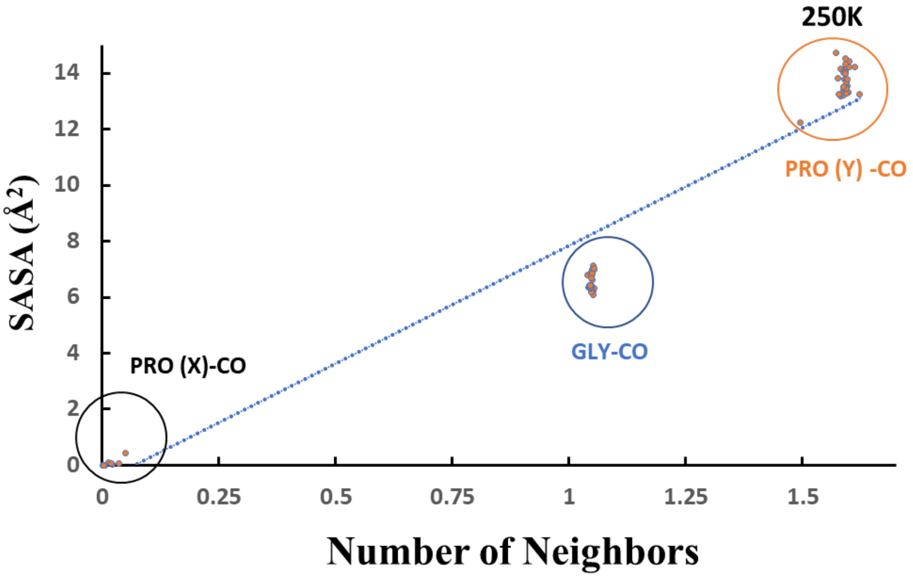

Figure S13: Correlation plot of the number of water neighbors with the oxygen carbonyl SASA values in our simulations of collagen triple-helix at 250 K [AMBER14SB+TIP4P/2005, 62306 WMs, 100 ns trajectory (10000 frames)]. To be compared with Figure 10 at 300 K.

Tcl Scripts.

Tcl script: S1. Average number of neighbors for each atom in a given selection

```
# Noam Agmon, HUJI, March 2022

# This Tcl script is designed to find and list neighbors of a given selection (using the VMD atom selection language)

# Example for usage: neighbors "resname Gly and name O" "output.dat"


proc neighbors {select output} {

            set outfile [open $output w]

            puts $outfile "index ave-neighbors"

            set sel [atomselect top $select frame 0]

            set selist [$sel list]

# get the atomindex from the list and calc its average number of neighbors

             foreach atomindex $selist {

                     set neb [neighbor $atomindex]

                      puts $outfile "$atomindex $neb"

                      puts stdout "$atomindex $neb"

             }


  close $outfile

}


proc neighbor {atomindex} {

# Number of water neighbors within r_min of an atom of given index, averaged over all frames

#input: minimum in g(r) for TIP4P water

           set r_min 3.225

#main loop over frames

    set nf [molinfo top get numframes]

            set neighbor 0

                     # loop over frames

  for {set i 0} {$i < $nf} {incr i} {

# saving the previous index and getting a new one
```

```
            set oldneighbor $neighbor
    set s2 [atomselect top "(water and oxygen) and within $r_min of index $atomindex" frame $i]
            set neighbor [$s2 num]
# This gets the number of water O atoms within r_min of selected atom
            set neighbor [expr {$oldneighbor + $neighbor}]
  }
  set neighbor [expr {($neighbor + 0.0) / $nf}]
```

Tcl script: S2 Calculate SASA for each residue or atom of a selection

```
# Satyaranjan Biswal,HUJI  July 2022
# This Tcl script is designed to calculate the Solvent Accessible Surface Area (SASA)
# For each residue in a specified selection of a molecular simulation trajectory. (The script is intended for use with VMD software)
# The output, which includes the average SASA for each residue, is written to a file.
# Example usage: sasa_resid "resname GLY" (For each residue)
# Example usage: sasa_resid "resname GLY and name O" (For each residue specified atom)

#####################################################
proc sasa {selection} {
# selection
set fr [open "SASA_$selection.dat" w]
set nf [molinfo top get numframes]
puts $fr "\n Total Frames found : $nf \n"
  set prt [atomselect top "protein"]
  set allsel [atomselect top $selection]
  # get list of residues
  set sellist [lsort -integer -unique [$allsel get index ]]
  #set sellist [lsort -integer -unique [$allsel get resid ]]
  ##setting sasa for every residue to zero
  foreach r $sellist {
  set resSASA($r) 0.0
```

```
#puts "setting 0.0 for residue $r"
  }
  for {set i 0} {$i <= $nf} {incr i} {
  $allsel frame $i
  $prt frame $i
  #$sellist frame $i
  $allsel update
  $prt update
  #$sellist update
  foreach r $sellist {
  #set sel1 [atomselect top "resid $r" frame $i]
  set sel1 [atomselect top "index $r" frame $i]
  set rsasa [measure sasa 1.4 $prt -restrict $sel1]
  #set user value for this frame
  #set rsasa [measure sasa 1.4 $sel1]
  #$sel1 set user $rsasa
  #$sel1 delete
  set sum 0.0
  foreach {tmp sum} [split [array get resSASA $r] ] break
 #puts "residue $r, sasa; $rsasa, old: $sum"
  set resSASA($r) [ expr { $sum + $rsasa } ]
# puts "$r resSASA is $resSASA($r)"
  } }
  foreach r $sellist {
  foreach {tmp sum} [split [array get resSASA $r] ] break
# puts $fr "$r, sasa: $sum"
}
  #set AvgSASA 0.0
  foreach r $sellist {
  foreach {tmp sum} [split [array get resSASA $r] ] break
  #-----------Avg over trajectory
  set AvgSASA($r) [ expr $sum/$nf ]
```

```
#puts $fr "Resid $r, Avg SASA: $AvgSASA($r)"
#puts $fr " $selection , Resid_atomic_index  $r, Avg SASA: $AvgSASA($r)"
#puts $fr " Resid_atomic_index  $r, Avg SASA: $AvgSASA($r)"
puts $fr " index  $r, Avg SASA: $AvgSASA($r)"
}
puts $fr "####################################################"
close $fr }
```

Tcl script: S3 Residence time and its distribution:

```
# Noam Agmon, HUJI, March 2022, November 2022
# (a) Residence time (in frames) of the closest particle from a selection (sel)
# on a given atomic site (atom) that hosts a single particle at a time
# replacement of a residing particle for a single frame is ignored
# output is a list of atoms and their residence times, "$index $res"
# (b) The residence time distribution in the interval [0, res_max], binned into N_d bins, where for the division: |---|---| we have N_d = 2
#  When averaging over several trajectories, the script should be executed with the same res-max and N_d values.
# sample input:
# residence-d "resid 6 and name O" "water and name OW and within 3.5 of resid 6" "res.out" "res_max" "N_d" "dist.out"
# Based on distance.tcl in VMD tutorial.pdf
proc residence-d {atom sel output1 res_max N_d output2} {
#main loop over frames
    set nf [molinfo top get numframes]
# Part (a) getting the residence time array and its average
        set outfile [open $output1 w]
        puts $outfile "atom = $atom , sel = $sel\n"
       #setting the stage at frame 1 of the loop, skipping frame 0
        set s1 [atomselect top "$atom" frame 1]
  set s2 [atomselect top "$sel" frame 1]
        set mindex [mind1 $s1 $s2]
```

```
set res 1
set total 0
# An empty array to hold the residence time vector:
array set resvec {}
# The initial length of the array
set nres 0
# loop over frames, checking at each step whether
#     the index of the closest particle has changed
for {set i 2} {$i < $nf} {incr i} {
set s1 [atomselect top "$atom" frame $i]
set s2 [atomselect top "$sel" frame $i]
# saving the previous index and getting a new one
set index $mindex
set mindex [mind1 $s1 $s2]
if {$mindex == $index} {
#increase the residence time by one unit frame and continues with the next for iteration
incr res
continue
}
# we get here only if the index changed in frame i
#       we check if it reverts in next frame, i+1
set i1 [expr {$i + 1}]
set s1 [atomselect top "$atom" frame $i1]
set s2 [atomselect top "$sel" frame $i1]
set mindex1 [mind1 $s1 $s2]
if {$mindex1 == $index} {
incr res
set mindex $index
# so a single frame change in index of the resident particle is ignored
# else write "$index $res"  and restart the count for a new residence event
} else {
puts $outfile "$index $res"
```

```
                set total [expr {$total + $res }]
                incr nres
                set resvec($nres) $res
                puts "$nres $index $res $total"
                set res 1
                        }
        }
        set meanres [expr {1.0 * $total / $nres}]
        puts $outfile "total = $total meanrestime = $meanres number restimes = $nres"
  close $outfile
  #################
# Part (b) calculate the restime distribution, in a new file
         set outfile [open $output2 w]
         #determine binsize, dr
         set dr [expr {1.0 * $res_max / $N_d }]
         puts $outfile "atom = $atom , sel = $sel , res_max = $res_max , N_d = $N_d, dr = $dr"
         puts "dr $dr"
         # set elements of the distribution array to zero
        for {set k 0} {$k < $N_d} {incr k} {
          set distribution($k) 0
        }
        # The distribution is obtained by looping over previously read frames
        #       adding 1 to bin k if resvec(k) is within the k'th bin
          for {set i 1} {$i <= $nres} {incr i} {
          set k [expr int($resvec($i) / $dr)]
          incr distribution($k)
          }
        # Write the distribution to file
        for {set k 0} {$k < $N_d} {incr k} {
                #puts $outfile "[expr ($k + 0.5) * $dr] $distribution($k)"
        }
        close $outfile
```

```
	parray distribution
	puts "done"
}

# A procedure for obtaining the index (mindex) of the closest particle
# (from a selection, sel) to a given atom
proc mind1 {atom sel} {
#Get the atom's coordinates
set ax [$atom get x]
set ay [$atom get y]
set az [$atom get z]
#Get the selection's list of coordinates
set sx [$sel get x]
set sy [$sel get y]
set sz [$sel get z]
set si [$sel get index]
#Set the minimal distance to the 1st atom in the selection
set minx [lindex $sx 0]
set miny [lindex $sy 0]
set minz [lindex $sz 0]
set mindex [lindex $si 0]
set mind [expr {sqrt (($ax - $minx) ** 2 + ($ay - $miny) ** 2 + ($az - $minz) ** 2)}]
#Iterate over the selection's list of coordinates
foreach x $sx y $sy z $sz index $si {
set distance [expr {sqrt (($ax - $x) ** 2 + ($ay - $y) ** 2 + ($az - $z) ** 2)}]
if {$distance < $mind} {
set mind $distance
set mindex $index }
}
# Output the selection index achieving minimal distance from atom
return $mindex
}
```

Tcl script: S4 RMSD: Measures average RMSD & std for given selections in a trajectory

```
# Satyaranjan Biswal,HUJI  July 2022

# This Tcl script is designed to calculate the Root Mean Square Deviation (RMSD). (The script is
intended for use with VMD software)

# This script returns both the average RMSD and its standard deviation across the trajectory.

# Example usage: rmsd "backbone" "top" 1

# Example usage: rmsd "protein" "top" "top"


proc rmsd {sel molid1 molid2} {
   # Get the number of frames in the first molecule
   set n [molinfo $molid1 get numframes]
   # Open the output file for writing
   set outfile [open "RMSD.dat" w]

   # Print input information to the output file and console
   puts $outfile "Given Input:\n=================="
   puts $outfile "Selection: $sel\nMolecule ID 1: $molid1\nMolecule ID 2: $molid2\n"
   puts $outfile "Total Frames: $n\n\n"
   puts "Given Input:\n=================="
   puts "Selection: $sel\nMolecule ID 1: $molid1\nMolecule ID 2: $molid2\n"
   puts "Total Frames: $n\n\n"

   # Initialize variables
   set sum 0
   set rmsd_list {}

   # Cycle through each frame
   for {set i 0} {$i < $n} {incr i} {
      # Select atoms in the two molecules
      set selA [atomselect $molid1 $sel frame $i]
      set selB [atomselect $molid2 $sel frame $i]
```

```
    # Align molecule B onto molecule A using the 'measure fit' command
    set transform_matrx [measure fit $selB $selA]
    $selB move $transform_matrx

    # Calculate the RMSD of the aligned molecules
    set rmsdAB [measure rmsd $selA $selB weight mass]
    # Print the RMSD value for the current frame to the output file and console
    puts [format "Frame: %5d     RMSD: %7.2f" $i $rmsdAB]
    puts $outfile [format " %5d    %7.2f" $i $rmsdAB]
    # Add the RMSD value to the list for later use
    lappend rmsd_list $rmsdAB
   # Accumulate the RMSD values for calculating the average RMSD
    set sum [expr $sum + $rmsdAB]
}

# Calculate the average RMSD
set avg_rmsd [expr $sum / $n]

# Calculate the standard deviation of the RMSD values
set sum_of_sq_diffs 0
foreach rmsd $rmsd_list {
    set diff [expr $rmsd - $avg_rmsd]
    set sum_of_sq_diffs [expr $sum_of_sq_diffs + $diff*$diff]
}
set std_rmsd [expr sqrt($sum_of_sq_diffs / ($n-1))]

# Print the average RMSD, standard deviation, and closing messages to the output file and console
puts [format "\n\nAvg. RMSD: %7.2f" $avg_rmsd]
puts [format "Std. Deviation: %7.2f\n\n" $std_rmsd]
puts "Data has been saved to RMSD.dat."
puts $outfile [format "\n\nAvg. RMSD: %7.2f" $avg_rmsd]
puts $outfile
```

Tcl script: S5 RMSF: Measures RMSF (Root Mean Square Fluctuation) in a trajectory

Satyaranjan Biswal,HUJI July 2022

This Tcl script is designed to calculate the Root Mean Square Fluctuation (RMSF). (The script is intended for use with VMD software)

RMSF provides a measure of the positional fluctuation of individual atoms (relative to a reference structure over a simulation).

Example usage: rmsf "name CA and protein" "1" (e.g., the C-alpha atoms of a protein and specify the molecule ID for which RMSF should be computed).

```
proc rmsf {sel molid} {
    # Get the number of frames in the molecule
    set n [molinfo $molid get numframes]

    # Create a list to hold the squared deviations for each atom
    set sqdevs [list]

    # Cycle through each frame
    for {set i 0} {$i < $n} {incr i} {
        # Select atoms in the molecule for the current frame
        set selA [atomselect $molid $sel frame $i]

        # Calculate the center of mass for the selected atoms
        set comA [measure center $selA]

        # Cycle through each selected atom
        foreach atom [$selA get index] {
            # Get the coordinates of the atom in the current frame
            set coords [lindex [$molid get coords $atom] $i]

            # Calculate the squared deviation of the atom's position from the center of mass
            set sqdev [expr {pow($coords(0)-$comA(0),2) + pow($coords(1)-$comA(1),2) +
pow($coords(2)-$comA(2),2)}]

            # Add the squared deviation to the list
```

```
            lappend sqdevs $sqdev
        }
    }

    # Calculate the average squared deviation for each atom
    set avg_sqdevs [lmap s [lrange $sqdevs 0 end] {expr {double($s)/$n}}]

    # Calculate the RMSF for each atom
    set rmsfs [lmap s $avg_sqdevs {expr {sqrt($s)}}]

    # Return the list of RMSF values
    return $rmsfs
}
```

```
Input: set sel "name CA and protein"
set molid 1
set rmsfs [rmsf $sel $molid]
```

Tcl script: S6 Radius of gyration (without the mass scaling)

```
# Satyaranjan Biswal,huji Feb 2023
# This Tcl script calculates the radius of gyration for a molecular simulation trajectory using VMD software. (without atomic mass scaling)
# Example usage:gyration "protein"
proc gyration {sel_string} {
    # Open an output file named "gyration.out" in write mode.
    set outfile [open "gyration.out" w]

    # Retrieve the number of frames in the trajectory of the top molecule.
    set nf [molinfo top get numframes]

    # Initialize variables to store the cumulative radius of gyration and a list to store the radius of gyration for each frame.
    set total_Rg 0
```

```
set Rg_list {}

# Select the atoms as specified by the user's input selection string.
set sel [atomselect top $sel_string]

# Loop through every other frame in the trajectory.
for {set i 0} {$i < $nf} {incr i 2} {
    # Update the frame for the selected atoms.
    $sel frame $i

    # Compute the radius of gyration for the current frame.
    set Rg [measure rgyr $sel]

    # Write the frame number and the computed radius of gyration to the output file.
    puts $outfile "$i,$Rg"

    # Store the computed radius of gyration in the Rg_list for later calculations.
    lappend Rg_list $Rg

    # Update the cumulative radius of gyration.
    set total_Rg [expr {$total_Rg + $Rg}]
}

# Compute the average radius of gyration over the frames.
set avg_Rg [expr {$total_Rg / ($nf / 2)}]

# Calculate the standard deviation of the radius of gyration values.
set sum_of_sq_diffs 0
foreach Rg $Rg_list {
    set diff [expr {$Rg - $avg_Rg}]
    set sum_of_sq_diffs [expr {$sum_of_sq_diffs + $diff*$diff}]
}
```

```
    set std_Rg [expr {sqrt($sum_of_sq_diffs / ($nf/2 - 1))}]

    # Write the average radius of gyration and its standard deviation to the output file.
    puts $outfile "Average radius of gyration: $avg_Rg"
    puts $outfile "Standard deviation: $std_Rg"
    # Notify the user that the data has been saved to the output file.
    puts "Data has been saved to gyration.out."
    # Close the output file.
    close $outfile
}
```

Tcl script: S7 Measures distance, average distance & std, for any given 2 atoms

```
# Satyaranjan Biswal,HUJI  Feb 2023
# Tcl script to measure the distance, average distance, and standard deviation between any given two atoms in a molecular simulation trajectory using VMD software.
# The script is particularly useful for tracking the end-to-end distance of proteins during a simulation by specifying the indices of the first and last atoms of each protein chain.
# Procedure Usage:calc_distance <selection1> <selection2>
# Where <selection1> and <selection2> are atom selection strings (e.g., "resid 3 and name CA").
# The function will calculate the distance between these two atoms for each frame in the trajectory.
# Example usage:# To compute the distance between the C-alpha atoms of residues 2 and 30 across a trajectory: calc_distance "resid 2 and name CA" "resid 29 and name CA"

proc calc_distance {sel1 sel2} {
    # Select the two atoms of interest
    set atom1 [atomselect top $sel1]
    set atom2 [atomselect top $sel2]

    # Get the number of frames in the trajectory
    set n [molinfo top get numframes]
    # Open the output file for writing
    set outfile [open "distances.dat" w]
```

```
    # Calculate the distance between the two atoms for each frame and write to output file
    set dist_list {}
    for {set i 0} {$i < $n} {incr i} {
        # Set the current frame of the atom selections
        $atom1 frame $i
        $atom2 frame $i
     # Calculate the distance between the two atoms
        set dist [measure bond $atom1 $atom2]
       # Write the frame number and distance to the output file
        puts $outfile "$i $dist"
        # Add the distance to the list for calculating the standard deviation
        lappend dist_list $dist
    }
    # Close the output file
    close $outfile
   # Calculate the average and standard deviation of the distances
    set avg_dist [expr {[tcl::mathop::sum $dist_list] / [llength $dist_list]}]
    set sum_of_sq_diffs 0
    foreach dist $dist_list {
        set diff [expr {$dist - $avg_dist}]
        set sum_of_sq_diffs [expr {$sum_of_sq_diffs + $diff*$diff}]
    }
    set std_dist [expr {sqrt($sum_of_sq_diffs / [expr [llength $dist_list] - 1])}]
    # Delete the atom selections
    $atom1 delete
    $atom2 delete
    # Return the average and standard deviation of the distances
    return [list $avg_dist $std_dist]
}
# Print the results
puts "Average distance: [lindex $result 0] angstroms"
puts "Standard deviation: [lindex $result 1] angstroms"
```
